# Infection risks associated with the 2022 FIFA World Cup in Qatar

**DOI:** 10.1016/j.nmni.2022.101055

**Published:** 2022-11-22

**Authors:** Jaffar A. Al-Tawfiq, Philippe Gautret, Patricia Schlagenhauf

**Affiliations:** Infectious Disease Unit, Specialty Internal Medicine, Johns Hopkins Aramco Healthcare, Dhahran, Saudi Arabia; Division of Infectious Diseases, Indiana University School of Medicine, Indianapolis, IN, USA; Division of Infectious Diseases, Johns Hopkins University, Baltimore, MD, USA; Aix Marseille Univ, IRD, AP-HM, SSA, VITROME, Marseille, France; IHU-Méditerranée Infection, Marseille, France; WHO Collaborating Centre for Travellers' Health, Institute for Epidemiology, Biostatistics and Prevention, University of Zürich Centre for Travel Medicine, MilMedBiol Competence Centre, University of Zürich, Switzerland

**Keywords:** FIFA World Cup, Mass gatherings, Qatar

The FIFA World Cup, a football tournament that attracts global attention, will be held in Qatar from November 20th to December 18th 2022. Teams and spectators from all continents will converge for this sporting spectacle. Apart from “football fever” what are the potential infectious disease risks for the players, the fans, the local hosting population and the countries of origin of the teams. To look at the spectrum of possible infectious disease risks, it is important to see the context of this event as a Mass Gathering (MG). There are two main types of MGs: planned or unplanned MGs which could be recurrent or spontaneous [1]. These events require a well conducted risk assessment and advanced planning to avoid the spread of emerging and endemic infectious diseases [2]. The most studied MGs is the annual Hajj and Umrah in Saudi Arabia [2,3]. And best examples of risk assessments are those conducted in 2020 and 2021 with delays of the Olympic games and the significant reduction in the number of pilgrims at the Hajj and cancelling Umrah during the early phases of COVID-19 pandemic [[4], [5], [6]]. But with the development of effective vaccines and significant decline of the global number of COVID-19 cases a gradual re-escalation of such events took place [4,7].

The FIFA World Cup in the last two decades took place in Japan and South Korea (2002), Germany (2006), South Africa (2010), Brazil (2014), and Russia (2018). This sporting event is one example of recurring mass gatherings that occur at different places around the world and an epidemiological look at infectious disease risks in geographic and temporal context can be useful [8]. Like other MGs, the FIFA World Cup unavoidably poses potential infectious disease risks to the host country (Qatar) and also to neighboring countries and other countries due to the risk of importation and subsequent exportation and also local acquisition of infectious diseases. MGs had been associated with the occurrence of outbreaks of infectious diseases particularly viral respiratory infections.

Qatar will host the FIFA World Cup from November 20 to December 18, 2022. Qatar is one of the six Gulf Cooperation Council (GCC) states with a population of 2.8 million, and expects to receive 1.2 million international visitors [9]. The FIFA World Cup 2022 will be hosted at the time when two Public Health Emergencies of International Concern (PHEIC) are concurrent. These are the COVID-19 pandemic and the monkeypox outbreak 2022. With respect to COVID-19, the number of the cases in Qatar continued to be reported at an average of 321 daily cases in November 2022 (Fig. 1). The emergence of the SARS-CoV-2 infection in 2019 resulted in another sports MG, the Tokyo 2020 Olympics, being postponed for 2021 [6] and had a major impact on annually occurring MGs such as the Hajj with subsequent gradual escalation of the Hajj pilgrimage [4,5,10]. There are limited data on the occurrence of respiratory tract infection outbreaks during sports events such as the Olympics [6]. For example, during the Pyeong Ghang Winter Olympics in South Korea, respiratory tract illnesses were the most common cause of illness [11,12]. This is in contrast to the well-studied Hajj pilgrimage with multiple studies showing respiratory tract infections as the major infectious diseases [[13], [14], [15]]. The Qatar ministry of health (QMoH) had released COVID-19 guidance and indicated that “currently there will be no vaccination requirement” [16]. Visitors are also not required to have pre-departure SARS-CoV-2 testing. The availability of effective COVID-19 vaccines and boosters should be utilized by visitors to Qatar to prevent the occurrence of COVID-19 during mass gatherings, at least in at risk attendees. However, the emergence of variants of SARS-CoV-2 for which vaccine efficacy might be reduced, is seen as a major threat to ending the COVID-19 pandemic and points to the occurrence of outbreaks in MGs. Previous successful Qatari experience in organizing a major football match held outside during the pandemic (Amid Cup Footbal Final of Qatar) under strict control is reassuring [17]. Another possible respiratory tract illness is the Middle East Respiratory Syndrome Coronavirus (MERS-CoV). MERS-CoV had caused multiple hospital outbreaks in Saudi Arabia [18] and had caused limited number of cases in Qatar and the pattern was sporadic [19]. Epidemiologic data from Qatar showed the occurrence of of 28 cases of MERS (incidence of 1.7 per 1,000,000 population) and most cases had a history of contact with camels [20]. Thus, people with greater risk of developing severe disease are advised to avoid contact with dromedary camels, drinking raw camel milk or camel urine, or eating meat that has not been properly cooked [21].

**Fig. 1:**
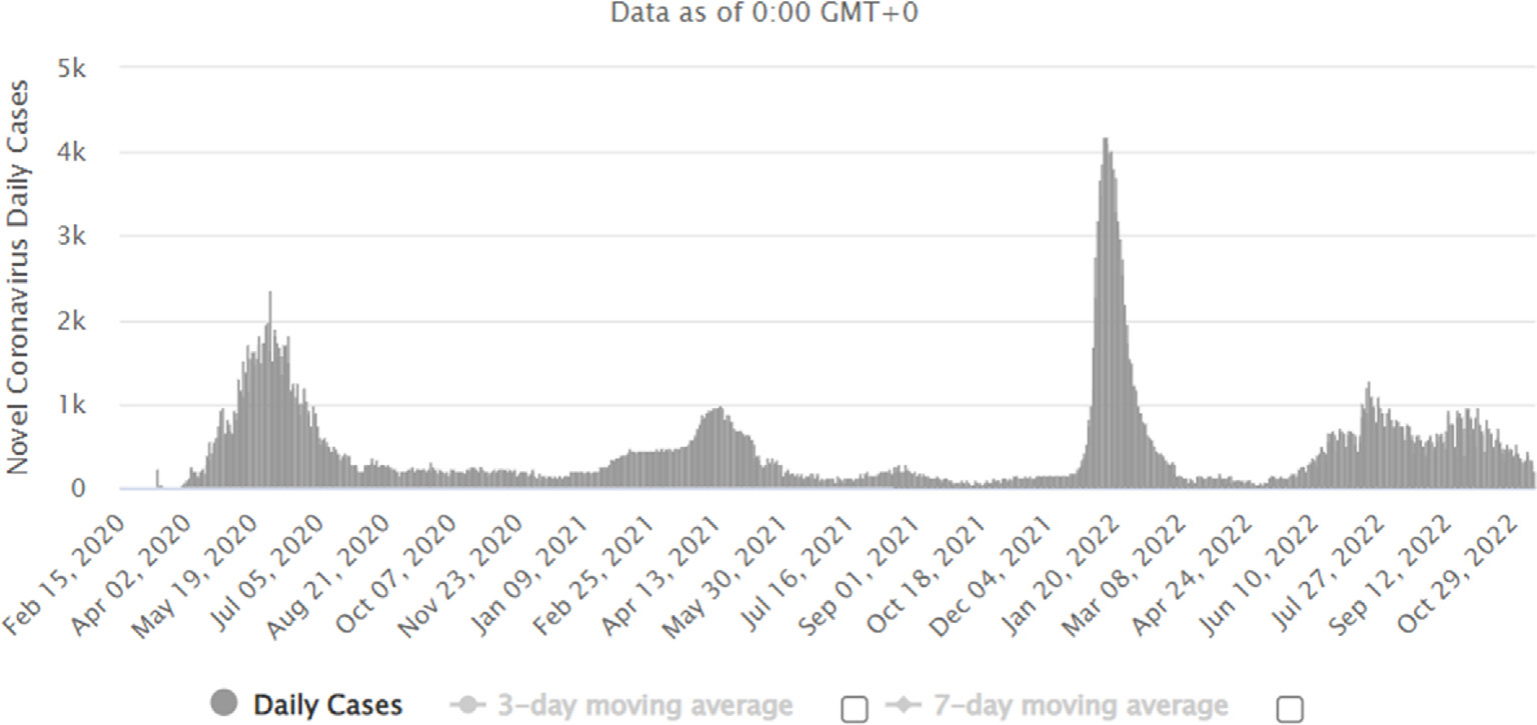
Daily New Cases of COVID-19 in Qatar.

Another infectious disease risk challenge at this time, is the occurrence of a multi-state monkeypox virus outbreak around the globe with the potential implication for MGs [22]. The number of reported cases to WHO is 79,641 as of 16 Nov 2022 [23]. One major difficulty with this virus is the difficulty in rapid detection of suspected cases, isolation of infected individuals and management of cases and contacts especially in large uncontrolled crowds [22]. To date, the State of Qatar had not reported any cases of monkeypox. However, in the neighboring countries there had been limited number of cases (8 cases in Saudi Arabia, and 16 cases in United Arab Emirates) (Fig. 2) [24]. The main transmission mode of the disease in the current outbreak is through close contacts, including notably sexual relations and the respiratory route plays a less important role if any [25]. Thus, it is important to avoid situations that put the individuals at risk of acquisition of monkeypox.

**Fig. 2:**
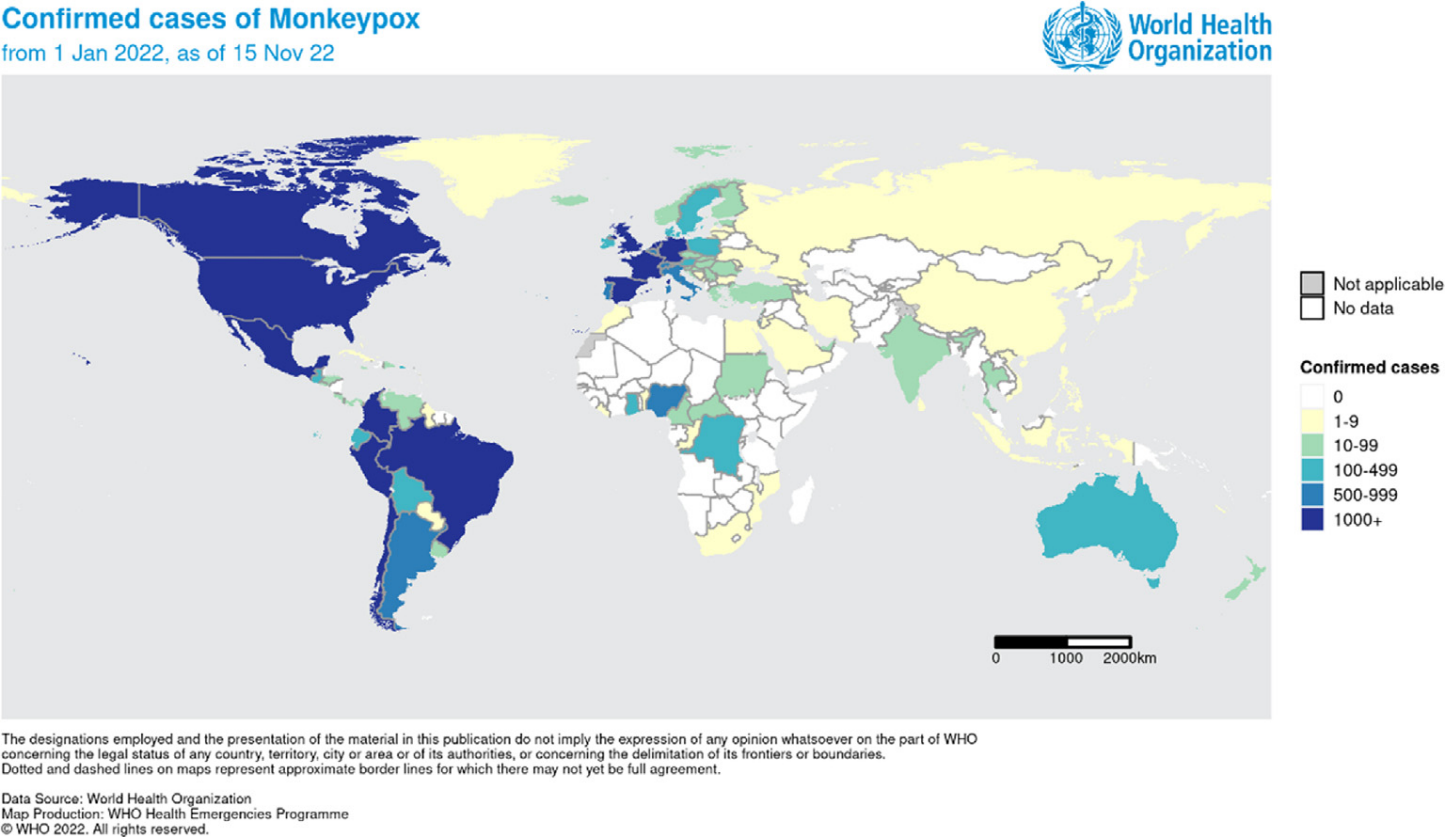
Confirmed Monkeypox Cases per the WHO [23] https://www.amhsr.org/articles/fifa-world-cup-2022-in-qatar-health-advice-and-safety-issues-for-travelling-attendees.pdf.

The occurrence of vector-borne diseases in Qatar is rare. For example, cutaneous leishmaniasis was previously a common disease in the neighboring Al-Hasa region, Saudi Arabia [26], autochthonous cases of cutaneous leishmaniasis had not been reported from Qatar [27]. Other vector-borne diseases such as malaria had not been reported in Qatar. Dengue incidence is low in Qatar and mostly reported in migrants [28]. In Qatar, *Ae. aegypti* was reported in 1999, but not in recent surveys, suggesting no risk of local transmission in the country. In 2018, a fox and a camel were found rabid in Qatar demonstrating that rabies virus is still present in Qatar, at least in rural areas [29]. Other possible infectious disease risks include measles, hepatitis A and B, travellers' diarrhoea and possible acquisition of multi-drug resistant bacteria (MDR) [30], with possible dissemination of MDR bacteria in MG [31]. To mitigate the afore-mentioned risks, visitors to the tournament should be up to date with their routine vaccinations and observe the rules for safe consumption of food and drinks.

In conclusion, the infectious disease risks associated with the FIFA World Cup 2022 this year in Qatar are dominated by the global concern about the ongoing COVID-19 pandemic with emergence of new variants and the threat of vaccine escape [32,33] and the occurrence of multi-state outbreak of monkeypox. Although in recent months, the trajectory of monkeypox cases points to decreasing numbers, this risk is still a significant challenge in the context of a football World Cup and possible sexual encounters. Qatar, the hosting country, had made the health sector in the country ready for such occurrence. Continued surveillance and studies of the effect of MGs on the transmission of infectious disease continue to be an important aspect of MGs. Novel technologies such as illness tracking Apps can [34] be considered for this and other large sporting and cultural events and should be employed to provide useful data for future MGs and enable recommendations for infectious disease prevention.

## Funding

This work received no funding.

## Ethical approval

Not applicable.

## Declaration of competing interest

The authors declare that they have no competing interests. JAT is an associate editor of NMNI and PS is EIC, NMNI.
